# Metformin Treatment Leads to Increased HIV Transcription and Gene Expression through Increased CREB Phosphorylation and Recruitment to the HIV LTR Promoter

**DOI:** 10.14336/AD.2023.0705

**Published:** 2024-04-01

**Authors:** Sahar Rezaei, Khalid A Timani, Johnny J He

**Affiliations:** ^1^Department of Microbiology and Immunology, Rosalind Franklin University, Chicago Medical School, North Chicago, IL 60064, USA.; ^2^Center for Cancer Cell Biology, Immunology and Infection, Rosalind Franklin University, North Chicago, IL 60064, USA.; ^3^School of Graduate and Postdoctoral Studies, Rosalind Franklin University, North Chicago, IL 60064, USA.

**Keywords:** Metformin, HIV transcription and replication, transactivation, gene expression, CREB phosphorylation, HIV LTR promoter

## Abstract

Antiretroviral therapy has effectively suppressed HIV infection and replication and prolonged the lifespan of HIV-infected individuals. In the meantime, various complications including type 2 diabetes associated with the long-term antiviral therapy have shown steady increases. Metformin has been the front-line anti-hyperglycemic drug of choice and the most widely prescribed medication for the treatment of type 2 diabetes. However, little is known about the effects of Metformin on HIV infection and replication. In this study, we showed that Metformin treatment enhanced HIV gene expression and transcription in HIV-transfected 293T and HIV-infected Jurkat and human PBMC. Moreover, we demonstrated that Metformin treatment resulted in increased CREB expression and phosphorylation, and TBP expression. Furthermore, we showed that Metformin treatment increased the recruitment of phosphorylated CREB and TBP to the HIV LTR promoter. Lastly, we showed that inhibition of CREB phosphorylation/activation significantly abrogated Metformin-enhanced HIV gene expression. Taken together, these results demonstrated that Metformin treatment increased HIV transcription, gene expression, and production through increased CREB phosphorylation and recruitment to the HIV LTR promoter. These findings may help design the clinical management plan and HIV cure strategy of using Metformin to treat type 2 diabetes, a comorbidity with an increasing prevalence, in people living with HIV.

## INTRODUCTION

Antiretroviral therapy (ART) has effectively suppressed human Immunodeficiency virus (HIV) replication and significantly prolonged the lifespan of HIV-infected population [1, 2]. However, this population has still faced other health-relevant disorders and complications. These include HIV-associated neurocognitive disorders [3-6], hypertension and cardiovascular diseases [7-10], renal impairment [11, 12], lipodystrophy [13-17], dyslipidemia [18, 19], premature/rapid aging [7, 20-22], insulin resistance, and diabetes mellitus [23-25]. The main attributing factors are lifestyle, host factors, HIV-related inflammation, and ART [26-34]. The incidence of type 2 diabetes is higher among HIV-infected individuals, as ART is often linked to insulin resistance and metabolic dysfunction [23, 35-39].

Metformin is the front-line medication of choice for treating type 2 diabetes and is being prescribed for approximately 120 million individuals worldwide [40]. Its anti-hyperglycemic action is primarily an outcome of decreased glucose production (without overt hypoglycemia) from the liver through inhibition of gluconeogenesis [40, 41] and increase in glucose uptake by voluntary/skeletal muscles, albeit to a less extent [42]. Metformin also improves insulin sensitivity by increasing the activity of insulin receptor through enhancing and lengthening the tyrosine phosphorylation of β-subunit of this receptor [43]. The mitochondrion is the primary target of Metformin, where it inhibits complex I of the mitochondrial electron transport chain, resulting in decreases in ATP and increases in 5’-adenosine monophosphate (AMP) and subsequent activation of AMP-activated protein kinase (AMPK). AMPK acts as a detector of cellular energy and a principal organizer of signaling pathways to maintain the balance between metabolic (lipid/glucose) and growth pathways and to restore cellular energy once the energy level is low [42, 44-48]. Furthermore, Metformin acts in a glucose control-independent manner by regulating plasma growth/differentiation factor 15, appetite, and weight in non-diabetic HIV individuals, further confirming its link to mitochondrial activities [49, 50].

The pleiotropic effects of Metformin have recently gained more attention, from improving lipid profiles [51, 52] and regulating inflammatory markers in either obese individuals with type 2 diabetes or experimental autoimmune encephalomyelitis animal models [53, 54] to ameliorating tumor establishment, progression, and cancer-related mortality rate [55, 56]. Metformin treatment has also been shown to impact HIV comorbidities such as lipodystrophy [13-17], cardiovascular diseases [57-59], and gut microbiota diversity [49, 50, 60] in HIV-infected individuals with or without type 2 diabetes. Several small clinical trials indicate possible effects of Metformin on HIV reservoirs in non-diabetic HIV-infected individuals who are treated by antiretroviral therapy for viral suppression [61-63]. However, whether and how Metformin itself affects HIV gene expression and replication is not known.

In the current study, we aimed to investigate the effects of Metformin on HIV gene expression and replication and the underlying molecular mechanisms. We began with introduction of HIV into the cells by transfection and infection, treated the cells with Metformin, and determined intracellular HIV gene expression and extracellular HIV production. We then determined the effects of Metformin on HIV gene transcription, expression of several major transcription factors, and the recruitment of these transcription factors to the HIV LTR promoter. We also determined effects of Metformin on HIV latency using latent cell lines. Lastly, we validated and substantiated the findings in HIV-infected human peripheral blood mononuclear cells (PBMC). All the results together demonstrated that Metformin enhanced HIV gene expression, transcription, and production and re-activated HIV from latency and that increased cAMP response element-binding protein (CREB) phosphorylation and expression and TATA-binding protein (TBP) expression and their recruitment to the HIV LTR promoter were likely involved in these processes.

## MATERIALS AND METHODS

### Cells, plasmids, transfection, and Metformin treatment

Human embryonic kidney epithelial cell line 293T was purchased from American Type Culture Collection (Manassas, VA). Jurkat clone E6-1 (#ARP-177) [64], HIV-1 lymphadenopathy-associated virus (LAV)-infected Jurkat E6 clone J1.1 (#ARP-1340), HIV-1 chronically infected U937 clone U1 (#ARP-165) and ACH-2 (#ARP-349) [65-67], HIV-1 LTR promoter-driven luciferase reporter cell line TZM-bl (#ARP-8129) [68-70], HIV-1 NL-4-3 LTR-driven luciferase reporter (#ARP-4788) [71, 72] were obtained through the NIH AIDS Reagent Program. NLGi latently infected Jurkat were generated by infecting Jurkat with NLGi and culturing the infected cells for over 63 days, changing the media every three days, and monitoring GFP expression in these cells. The Buffy coat was purchased from Versiti (Indianapolis, IN). 293T and TZM-bl were cultured in Dulbecco’s modified Eagle’s medium (Corning, Manassas, VA). Jurkat, PBMC, ACH-2, J1.1, U1, and NLGi latent Jurkat were cultured in RPMI 1640 medium (Corning). Both DMEM and RPMI 1640 were supplemented with 10% fetal bovine serum (R&D Systems, Minneapolis, MN), 100 U/ml penicillin/ 100 μg/ml streptomycin (Cat # P4333, Sigma-Aldrich, St. Louis, MO) and all cells were cultured in a 37°C, 5% CO_2_ incubator. 293T were transfected using the standard calcium phosphate precipitation method [73, 74], which often gives rise to 100% transfection efficiency. pcDNA3, pAP-1-Luc, and pNF-κB-Luc were from Clontech (Mountain View, CA). pGL3.TATA-Luc had the synthetic adenovirus E1b TATA sequence (TATATAAT) inserted in the pGL3 backbone (Promega, Madison, WI) [75]. pNL4-3 and pNL4-3-Luc-E- were described elsewhere [76]. HIV reporter virus vector NLGi, a derivative from the pNL4-3 HIV vector with the green fluorescent protein (GFP) reporter gene inserted preceding the Nef gene, was a gift from Dr. B. K. Chen of Mount Sinai School of Medicine [77]. HIV LTR core-Luc reporter was constructed by inserting the LTR core promoter extending upstream to contain the NF-κB DNA binding sites (from pNL4-3 construct) into the pGL3 backbone using the standard cloning technique with PCR primers 5’-TAG AGA GCT CTC TAC AAG GGA CTT TCC G-3’ and 5’-GAG ACA AGC TTT GCT TAT ATG CAG CAT CTG-3’. Lipofectamine™ 3000 Transfection Reagent (# L3000001, ThermoFisher Scientific, Waltham, MA) was used to transfect 293T with pAP-1-Luc, pNF-κB-Luc, and p-TATA-Luc. Metformin was purchased from Cayman Chemical (#13118, Ann Arbor, Michigan), freshly prepared in phosphate-buffered saline (PBS) and used to treat the cells as indicated. 666-15 was purchased from MilliporeSigma (# 5383410001, Burlington, MA), dissolved in dimethyl sulfoxide (DMSO), and added to the cells at an effective concentration as reported [78, 79].

### HIV production and infection

293T were plated in a 10 cm cell culture dish at a density of 2 x 10^6^ cells per dish and transfected with 20 μg pNL4-3 or 3.3 μg pVSV-G plus 16.7 μg pNL4-3-Luc-E- using the standard calcium phosphate precipitation method. The cells were cultured for 16 h, the culture medium was replaced with fresh medium, and continued to culture for 48 h. The culture medium was collected and briefly centrifuged to remove the cell debris. The cleared supernatant was passed through a 0.45 µm syringe filter (SIMSII, Issaquah, WA) and subjected to 20% sucrose ultracentrifugation at 100,000 x *g*, 4^o^C for 2 h. The virus pellet was suspended in PBS, and the suspended viruses were aliquoted, stored at -80^o^C, and used as virus stock. The virus titer was determined using the reverse transcriptase assay (RTase assay, see below). Jurkat were infected with NL4-3 by spinoculation at 850 x *g*, room temperature for 2 h in the presence of 8 µg/ml polybrene or transduced by VSV-G-pseudotyped NL4-3-Luc-E-. The infected cells were washed with fresh culture media after infection/transduction and processed for the subsequent experiments.

### Cell lysate preparation and Western blotting

Cells were washed twice with ice-cold PBS and lysed in RIPA buffer [10 mM Tris.HCl, pH 8.0, 1 mM EDTA, 0.5 mM EGTA, 0.1% sodium deoxycholate, 0.1% SDS, 140 mM NaCl, 1% Triton X-100, 1 mM PMSF, and 1X protease and phosphatase inhibitor cocktail (ThermoFisher Scientific)] on ice for 20 min. The cell debris was removed by centrifugation at 12,000 *x g* for 15 min to obtain the clear cell lysates. The protein concentration in the lysates was determined using a Bio-Rad DC protein assay kit (Bio-Rad, Hercules, CA) and a Bio-Rad iMark microplate reader. The proteins in the lysates were separated by sodium dodecyl sulfate-polyacrylamide gel electrophoresis (SDS-PAGE), transferred onto polyvinylidene difluoride membrane, and probed with a primary antibody and an appropriate secondary antibody. The WB membranes were visualized using enhanced chemiluminescence substrates (#32106, ThermoFisher Scientific) and imaged on a Chemidoc MP imaging system (Bio-Rad). The primary antibodies were rabbit monoclonal antibodies AMPKα (#2532), phospho-AMPKα (Th172) (#2535), CREB (#9197), phospho-CREB (Ser133) (#9198), CBP (#7389), p300 (#54062), TBP/TFIID (#44059) from Cell Signaling (Danvers, MA); mouse monoclonal antibodies GAPDH (#sc-32233) from Santa Cruz Biotechnology (Dallas, TX); Anti-HIV-1 p24 hybridoma (183-H12-5C, #ARP-1513) [80] and rabbit polyclonal anti-HIV-1 Nef protein (#ARP-2949) [81] from NIH AIDS Reagent Program; and mouse monoclonal antibody β-actin (#A1978) from Sigma-Aldrich (St. Louis, MO). The secondary antibodies HRP-conjugated sheep anti-mouse IgG (#NA931V) and HRP-linked donkey anti-rabbit IgG (#NA9340V) were from MilliporeSigma.

### RTase assay

The RTase assay was performed as previously described [82]. The supernatants containing viruses were collected 48 h post-transfection and spun down at 500 x *g* for 5 min to remove cell debris. The supernatants were then transferred to new tubes and centrifuged at 21,300 x *g*, 4°C for 1 h. The virus pellets were suspended and lysed in 10 µl dissociation buffer (50 mM Tris.HCl, pH 7.5, 0.25 M KCl, 0.25% Triton X-100, 20% glycerol, and 1 mM DTT), followed by subjecting the suspended pellets to three quick freeze-thaw cycles. Subsequently, a 40 µl reaction mixture including 34 µl RT assay buffer (50 mM Tris.HCl pH 7.5, 7.5 mM MgC1_2_, and 0.05 % Triton X-100, 0.5 mM DTT), 5 µl 1 mg/ml poly(A) x (dT)_15_ (Roche Diagnostics, Indianapolis, IN), and 1 µl [Methyl^3^H]-deoxythymidine 5’-triphosphate tetrasodium salt (Perkin Elmer, Waltham, MA) was added to the virus lysate and incubated at 37°C for 1 h. The reaction mixtures were then spotted on nitrocellulose membrane (Bio-Rad, Hercules, CA). The membranes were washed with 2X SSC buffer (0.3 M sodium chloride, 30 mM sodium citrate) three times, 5 min each, followed by rinsing the membranes in 100% ethanol. The membranes were then air-dried and counted for the ^3^H radioactivity in the scintillation counting fluid (#6013631, Perkin Elmer) on a microβeta2 scintillation counter (Perkin Elmer). The RTase activity was expressed as counts per min (CPM) per milliliter of the supernatant.

### Cell viability and proliferation assays

The 3-(4,5-dimethylthiazol-2-yl)-2,5-diphenyltetra-zolium bromide (MTT) assay was used to determine cell number and viability. 293T were seeded in a 24-well plate at a density of 0.6 x 10^5^ per well and cultured for 24 h. The cells were then transfected with pcDNA3 or pNL4-3. Media was replaced with fresh DMEM medium 16 h after transfection, and cells were treated with either PBS or various concentrations of Metformin for 48 h. TZM-bl and HIV-infected Jurkat cells were seeded at the same density as 293T above and cultured for 24 h and treated with PBS/Metformin for 48 h. The medium was removed and replaced with a complete DMEM medium for 293T and TZM-bl cells or RPMI 1640 medium for Jurkat. MTT (#298-93-1, Biosynth International, Inc, Itasca, IL) was added to each well to the final concentration of 1 mg/ml. The plate was incubated in the dark, 37°C for 4 h. The media containing MTT was removed, and DMSO was added to completely dissolve the formazan crystals while the plate was wrapped with aluminum foil and mixed by rocking on a shaker at room temperature for 20-30 min. The plate was briefly centrifuged. The supernatants (100 μl each) were transferred to a 96-well plate for optical density reading, which was performed at a wavelength of 595 nm with the reference wavelength of 655 nm using an iMark plate reader (Bio-Rad). The solvent DMSO was used as the background reading and subtraction. A fluorometric cell proliferation assay kit (#K307-1000, BioVision, Waltham, MA) was also used to determine the cell number. Briefly, HIV-infected Jurkat were seeded in a 96-well white tissue culture plate with a clear bottom at a density of 1 x 10^4^ per well and cultured for 24 h. The cells were treated and incubated with PBS or Metformin for 48 h. After incubation, prepared and well-mixed 5X nuclear dye/cell lysis buffer solution (25 µl) was added directly to each well. The plate was gently rocking on a shaker at room temperature for 15 min while being protected from light. The fluorescence of the cells was measured using a microplate reader (Biotek Synergy HT) at Ex/Em = 480/538 nm. The cell number was calculated using a standard curve obtained from serial dilutions of Jurkat with known cell counts.

### RNA isolation, semi-quantitative reverse transcription-polymerase chain reaction (RT-PCR), and quantitative real-time PCR (qRT-PCR)

Total RNA was extracted using the TRIzol RNA isolation reagent (#15596018, Invitrogen, Carlsbad, CA) according to the manufacturer’s instructions except for the inclusion of an additional step of acidic phenol extraction (#AM9722, Invitrogen) to prevent residual transfected plasmid DNA in the RNA from being PCR amplified. Total RNA was used to synthesize cDNA using an iScript™ Reverse Transcription Supermix (#1708890, Bio-Rad). The cDNA was subjected to the semi-quantitative PCR with the program of 1 cycle of 95°C for 3 min, 35 cycles of 95°C for 1 min, 55°C for 1 min, and 72°C for 2 min, and 1 cycle of 72°C for 8 min, or the qRT-PCR using SYBR Green mix (#1725270, Bio-Rad) with the program of 1 cycle of 95°C for 3 min and 40 cycles at 95°C for 15 sec, and 60°C for 1 min. The primers were P9501: 5’-CAG ATG CTG CAT ATA AGC AGC TG-3’ and 5T25: 5’-TTT TTT TTT TTT TTT TTT TTT TTT TTG AAG-3’ for total HIV RNA (unspliced and spliced) [83]; SK145: 5’-AGT GGG GGG ACA TCA AGC AGC CAT GCA AAT-3’ and SK39: 5’-TTT GGT CCT TGT CTT ATG TCC AGA ATG C-3’ for HIV gag-pol RNA (unspliced) [84, 85]; 5’-GAA ACT GTG GCG TGA TGG C-3’ and 5’- CCA GTG AGC TTC CCG TTC AG-3’ for GAPDH, an internal control for normalization. For semi-quantitative RT-PCR, we titrated and optimized the amount of input RNA for reverse transcription and cDNA for PCR to ensure that the amplification was in the linear range for comparison.

### Luciferase reporter gene assay

Cells were washed with ice-cold PBS and lysed with 1X passive lysis buffer (#E4030, Promega) for 15 min with intermittent mixing. The cleared cell lysates were obtained by brief centrifugation and added with firefly luciferase assay substrate (#E1500, Promega) at the ratio of 1:4 (5 µl sample plus 20 µl substrate). The luciferase activity was measured using a Lumat LB 9501 Single Tube Luminometer (Berthold, Hartford, CT). The protein concentration of the cleared cell lysates was also determined using a Bio-Rad DC protein assay kit (Bio-Rad) and used to normalize the luciferase activity, which was expressed as relative luminescence/light unit (rlu).

### Chromatin immunoprecipitation (ChIP) assay

The ChIP assay was performed according to the protocol established by Rockland Immunochemicals, Inc. (Pottstown, PA) with some modifications. Briefly, cells were washed with ice-cold PBS twice, added freshly made 5 mM dimethyl 3,3′-dithio-bis (propionimidate) dihydrochloride (#38285-78-8, Sigma-Aldrich), incubated on ice for 30 min, added ice-cold quenching buffer (100 mM Tris.HCl pH 8.0, 150 mM NaCl), and then washed with ice-cold PBS twice. The cells were then added 1% formaldehyde (#410730050 ThermoFisher Scientific), incubated at room temperature for 10 min, added the second quenching buffer (125 mM glycine), incubated at room temperature for 5 min, and washed with PBS twice. The cross-linked cells were suspended in the swelling buffer [25 mM Hepes, pH 7.8, 1.5 mM MgCl_2_, 10 mM KCl, 0.1% NP-40, 1 mM DTT, 0.5 mM PMSF, 1X protease and phosphatase inhibitor cocktail (#A32959, ThermoFisher Scientific)], followed by repeated pipetting and incubation on ice for 15 min. Nuclei were then pelleted and suspended in the nuclear lysis buffer [50 mM Hepes, pH 7.9, 140 mM NaCl, 1 mM EDTA, 1% Triton X-100, 0.1% sodium deoxycholate, 0.1% SDS, 0.5 mM PMSF, 1X protease and phosphatase inhibitor cocktail (#A32959, ThermoFisher Scientific)]. The nuclear lysates were then treated with 2.5 units/ml micrococcal nuclease (MN, #88216, ThermoFisher Scientific) in the MN reaction buffer (50 mM Tris.HCl, pH 8.0, 5 mM CaCl_2_) at room temperature for 10 min. The reaction was stopped with EGTA, pH 8.0 at a final concentration of 20 mM. The samples were then further sonicated using Sonic Dismembrator (Fisherbrand™ Model 505, Fisher Scientific, Pittsburgh, PA) on ice with 5-7 pulses, each for 10 sec, with the 10-sec intervals on ice between pulses, to break the nuclear membrane and facilitate the release of all the fragmented chromatin. Subsequently, the lysates were pre-cleared by adding 30 µl protein A agarose beads (#20333, ThermoFisher Scientific)/ml lysates and rotating the mixtures at 4°C for 1.5 h, followed by centrifugation of samples at 2000 x *g*, 4°C for 5 min to remove the beads, while the cleared nuclear lysates were saved and transferred to the new microcentrifuge tubes. The primary antibodies of 2-4 µg rabbit monoclonal antibodies for desired targets that were used for Western blotting above (Cell Signaling) or normal rabbit IgG (#J2909, Sigma), were added to the samples and incubated at 4ºC on a rotator overnight, followed by adding protein A agarose beads that were pre-incubated with BSA/Salmon Sperm DNA, and continued to incubate at 4ºC for 4 h. The beads were recovered by brief centrifugation and washed sequentially twice with each of the following buffers: Low-salt buffer (50 mM Tris.HCl, pH 8.0,150 mM NaCl, 0.1% SDS, 1% Triton X-100, 1 mM EDTA), High-salt buffer (50 mM Tris.HCl, pH 8.0, 500 mM NaCl, 0.1% SDS, 1% Triton X-100, 1 mM EDTA), LiCl buffer [50 mM Tris.HCl, pH 8.0, 250 mM LiCl, 1% sodium deoxycholate, 1% Nonidet P-40, 1 mM EDTA, 0.5 mM PMSF, protease inhibitor cocktail (#A32953, ThermoFisher Scientific)], and TE buffer (10 mM Tris.HCl, pH 8.0, 0.25 mM EDTA pH 8.0) [86]. Each wash was performed with rotation at 4ºC for 10 min. The agarose beads were recovered by brief centrifugation, and the DNA-protein complexes were eluted using elution buffer (50 mM Tris.HCl, pH 8.0, 1 mM EDTA pH 8.0, 1% SDS, 50 mM NaHCO_3_), followed by addition of 200 mM NaCl and treatment with 0.1 µg/µl DNase- and protease-free RNase A (#EN0531, ThermoFisher Scientific) at 65ºC overnight and then treatment with 0.2 µg/µl proteinase K (#MC5005, Promega, Madison, WI) at 42ºC for 2 h. The input enzymes and remaining cellular proteins were removed by phenol-chloroform extraction. Subsequently, DNA was recovered by ethanol precipitation (1 µl 20 µg/µl Glycogen; #R0561, ThermoFisher Scientific, 7.5 M NH_4_OAc in the amount of 0.5 X volume of sample and 100% ethanol in the amount of 2.5X volume of the sample) and used as the template for qPCR. The primers were 5’-CAT CCG GAG TAC TTC AAG AAC TGC-3’ and 5’-GGC TTA AGC AGT GGG TTC CCT AG-3’ for the 3’ LTR-promoter region (nt. 8984-9202) [86]; 5’-GAG CTT TCT ACA AGG GAC TTT C-3’ and 5’-AGA CCC AGT ACA GGC AAA-3’ for the 5’ LTR promoter region (nt. 337-459) [87]; and 5’-CTA GCA TTT CGT CAC ATG GCC-3’ and 5’-GTG GGT TCC CTA GTT AGC CAG-3’ for the larger portion of 5’ LTR region (nt. 276-514) targeting CBP, p300 along with TBP, and CREB, and phosphorylated CREB [88]. qPCR was also performed with primers 5’-GTG CTC GCT TCG GCA GCA CA-3’ and 5’-AAA ATA TGG AAC GCT TCA CGA-3’ for U6 to determine the input DNA for normalization.

### Human peripheral blood mononuclear cells (PBMC) isolation, activation, and infection

Human PBMC were isolated from fresh buffy coat collected from healthy donors using the density gradient centrifugation method. Briefly, buffy coat was diluted with 2X volume PBS and was gently layered on top of an equal volume Ficoll-Paque PLUS (#17144003, Cytiva, Marlborough, MA), followed by centrifugation at 400 x *g* for 40 min without break. The PBMC were removed and transferred to a new 50 ml tube, washed in 25 ml DPBS buffer twice, and recovered by centrifugation at 350 x *g* for 10 min. The cells were counted and cultured in the presence of 1 µg/ml anti-human CD3 antibody (#317302, BioLegend, San Diego, CA) and 2 µg/ml anti-human CD28 antibody (#302902, BioLegend) for 72 h. The cells were then infected with NL4-3 at a MOI as indicated in the presence of 8 µg/ml polybrene by spinoculation at 850 x *g*, room temperature for 2 h. The cells were recovered by centrifugation, washed with fresh media, and continued to culture in the presence of 100 IU/ml human IL-2 (#21-8029-U050, Tonbo Biosciences, San Diego, CA) and used in subsequent Metformin experiments.

### Statistical analysis

All data except for the ChIP assay results were analyzed by one-way ANOVA with equal variances and normal distribution followed by either *Bonferroni* or *Dunnett post hoc* tests unless stated otherwise. The results obtained from ChIP assay were analyzed using Two-way ANOVA. *: *p* < 0.05, **: *p* < 0.01, ***: *p* < 0.001.

## RESULTS

### Metformin treatment increased HIV production and intracellular HIV gene expression

**Figure 1.**
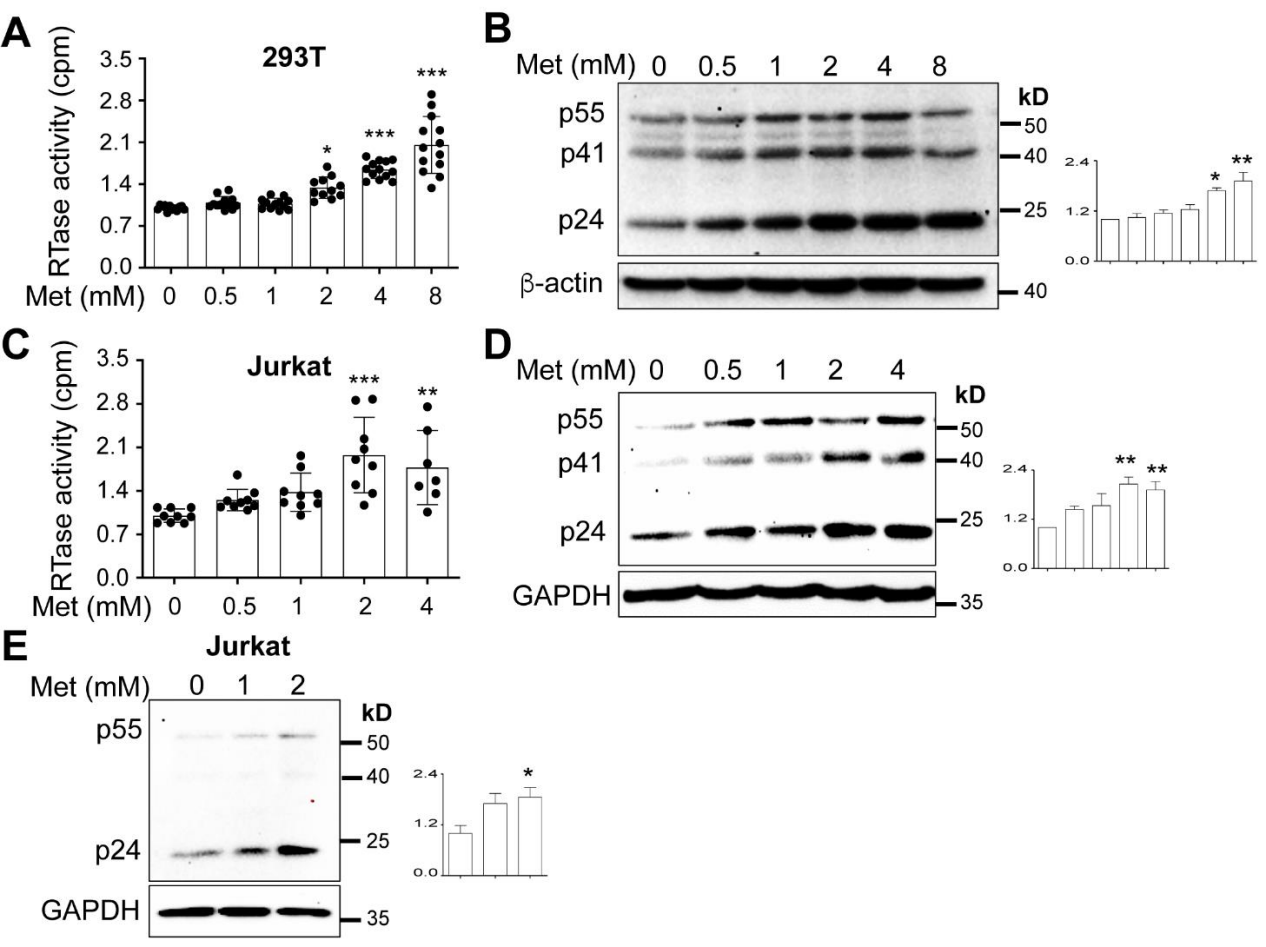
**Effects of Metformin on HIV production**. (A & B) 293T were plated in a 6-well cell culture plate at a density of 2 x 10^5^ per well, transfected with 3.5 μg pNL4-3, cultured for 16 h, changed medium, added Metformin, and continued to culture for 48 h. The culture medium was collected to determine HIV production by the reverse transcriptase (RTase) activity assay (A), while the cells were harvested to determine HIV intracellular gene expression by Western blotting against an anti-p24 antibody or anti-b-actin antibody (B). p24 expression was quantitated by densitometry, normalized to the loading control, b-actin, and expressed by fold-change in reference to the first sample without Metformin treatment. (C & D) Jurkat (1 x 10^6^) were infected with 10,000 cpm RT equivalent HIV NL4-3 viruses via spinoculation in the presence of 1 x polybrene, washed with PBS, cultured for 5 days with changes of fresh medium every 48 h to reach the maximum number of infected cells, added Metformin, and continued to culture for 72 h. The culture medium was collected to determine HIV production by the RTase assay (C), while the cells were harvested to determine HIV intracellular gene expression by Western blotting against an anti-p24 antibody or anti-GAPDH antibody (D). p24 expression was quantitated as above. **(E)** 1 x 10^5^ Jurkat cells were seeded in 12-well plate and infected with 100,000 cpm RT equivalent VSV-G-pseudotyped HIV-Luc, added Metformin, and cultured for 72 h. The cells were washed with PBS and collected for Western blotting against an anti-p24 antibody or anti-GAPDH antibody. p24 expression was quantitated as above. The RTase activities were normalized to the cell counts (A & C). The data were Mean ± SD of multiple samples (A, N = 12; C, N = 9) or representative of three to four independent experiments (B, D, & E).

To determine if Metformin treatment would alter HIV production, we first transfected 293T with pNL4-3 and treated the cells with Metformin, collected the culture supernatant, and determined the HIV level in the supernatant. We observed that Metformin increased HIV production beginning at a concentration of 0.5 mM and up to 8 mM (Fig. 1A). We also noticed significantly fewer cells when Metformin reached 8 mM (Supplementary Fig. 1A). Thus, we chose to use Metformin at the concentration of 0-4 mM for the subsequent experiments. To determine if Metformin would also increase intracellular HIV gene expression, we harvested the same transfected cells, prepared cell lysates, and performed Western blotting using an anti-p24 antibody as a marker for late gene expression and an anti-Nef antibody for early gene expression. There were parallel increases of p24 and its precursors p55/41 with more Metformin (Fig. 1B) and parallel increases of Nef expression with more Metformin (Supplementary Fig. 1B).

**Figure 2.**
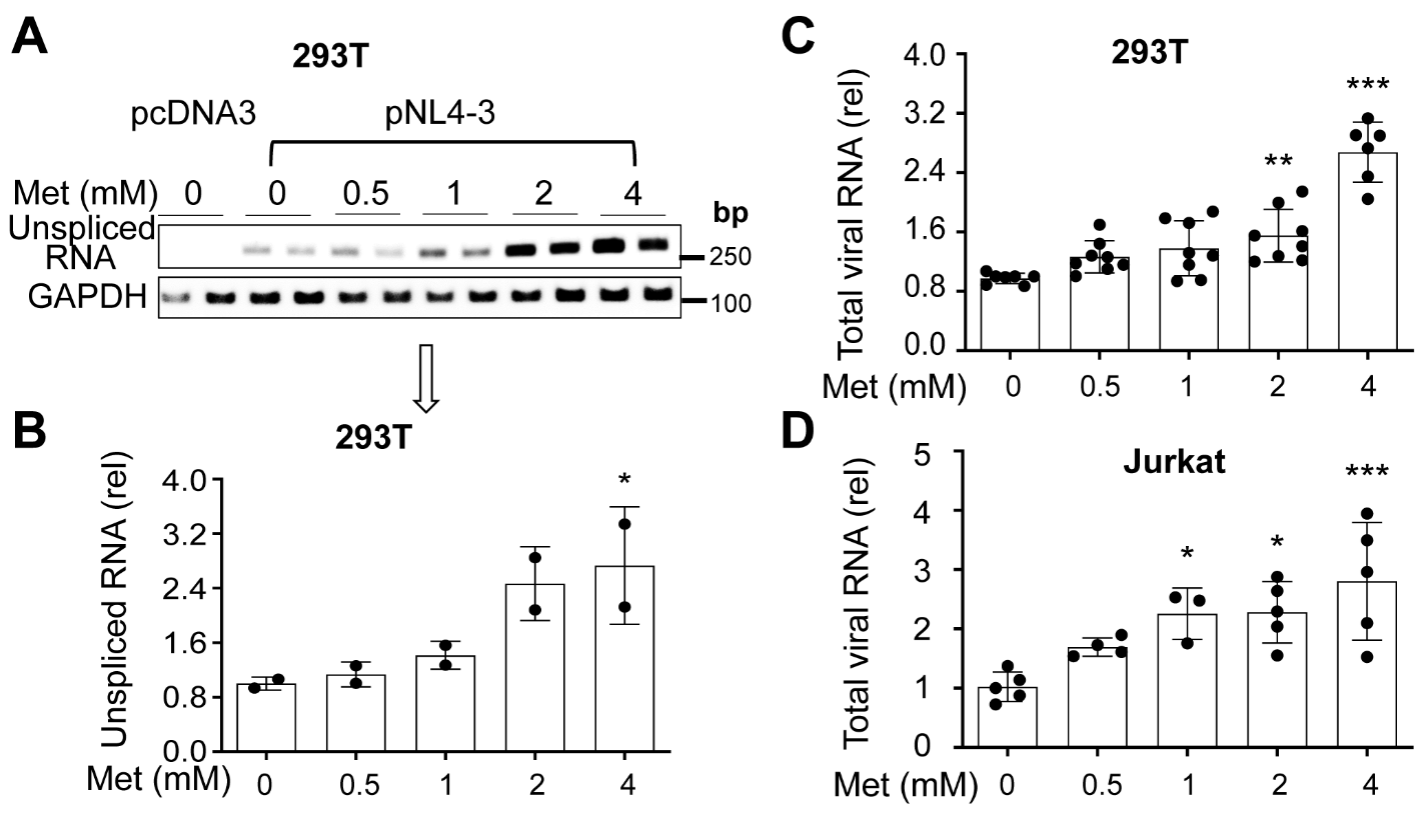
**Effects of Metformin on HIV RNA expression**. **(A-C)** 293T were plated in a 6-well cell culture plate at a density of 2 x 10^5^ per well, transfected with 3.5 μg pcDNA3, or pNL4-3, cultured for 16 h, changed medium, added Metformin, and continued to culture for 48 h. The cells were harvested for total RNA isolation and determined for gag-pol RNA by the conventional RT-PCR followed by agarose gel electrophoresis (A) and densitometry quantitation for unspliced gag-pol RNA (B) or determined for total viral RNA by real-time qRT-PCR (C). **(D)** Jurkat were infected with HIV NL4-3 viruses for 5 days as above, added Metformin, and cultured for 72 h. The infected cells were harvested for total RNA isolation and determined for total viral RNA by real-time qRT-PCR. The data were Mean ± SD of multiple samples (A & B, N = 2; C, N ≥ 6; D, N ≥ 3).

We next determined Metformin effects on HIV production and intracellular HIV gene expression in the context of HIV infection. To this end, we infected Jurkat with NL4-3, treated the cells with Metformin, collected the supernatant for HIV production, and harvested the cells for intracellular gene expression. Similar to the findings from the transfection, more HIV and more p24 and its precursor p55/41 were detected with more Metformin in the context of HIV infection (Fig. 1C & D). Similarly, cytotoxicity was detected with Metformin at a concentration of 1-2 mM or higher, determined by the cell viability assay or direct cell counting (Supplementary Fig. 2A-B). Thus, we decided to use 2 mM or lower Metformin for most subsequent experiments. Moreover, we used the total cellular protein or cell count to normalize the results in these experiments. To further confirm Metformin effects on HIV gene expression, we infected Jurkat with replication-defective VSV-G-pseudotyped HIV-Luc, treated the cells with Metformin, and performed Western blotting. Comparably, more p24 and its precursor p55 were detected in these cells with more Metformin (Fig. 1E). Taken together, these results demonstrated that Metformin treatment increased HIV production and intracellular HIV gene expression.

### Metformin treatment increased HIV RNA expression and transcription

**Figure 3.**
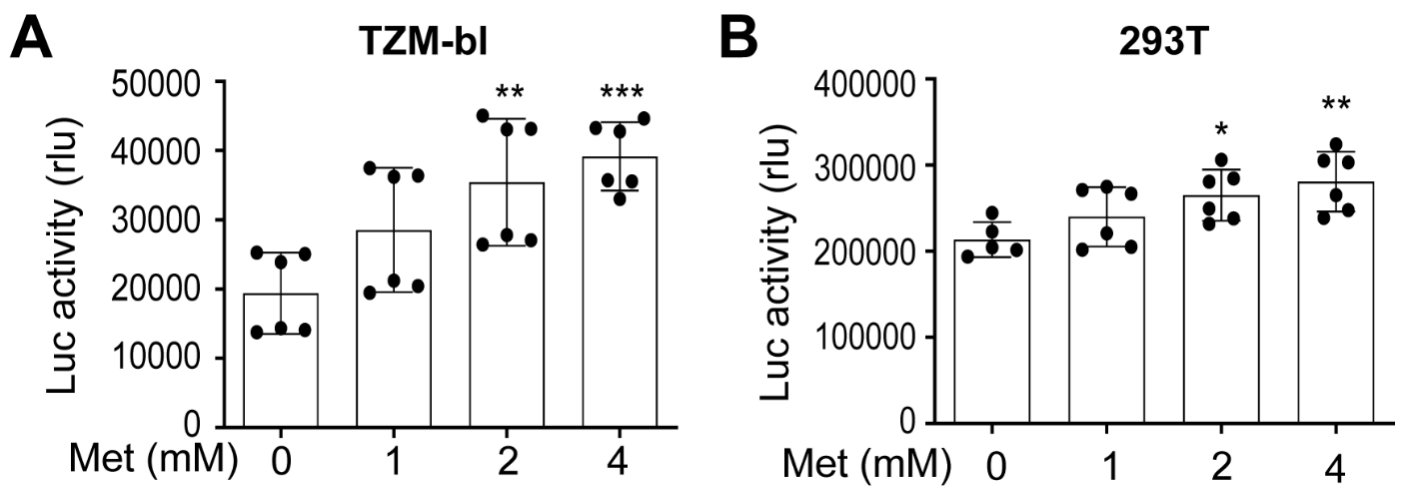
**Effects of Metformin on the HIV LTR promoter activity**. **(A)** TZM-bl were plated in a 12-well plate at a density of 1 x 10^5^ cells/well, added Metformin, cultured for 48 h, and harvested for the Luc reporter gene assay. **(B)** 293T were plated in a 12-well plate at a density of 1 x 10^5^ cells/well, transfected with 10 ng of HIV-LTR-Luc adjusted to 1.5 µg DNA per well using pcDNA3, cultured for 16 h, changed with fresh medium, added Metformin, continued to culture for 48 h, and harvested for the Luc reporter gene assay. The Luc activity was normalized to the corresponding cellular protein concentration or cell counts. The data were Mean ± SD of six samples (A & B, N = 6).

To determine if Metformin-enhanced HIV gene expression and production resulted from increases in HIV transcription, we determined the level of unspliced HIV RNA in HIV-transfected Metformin-treated 293T using the conventional semi-quantitative RT-PCR and a pair of gag-pol specific primers [84, 85]. More Metformin led to higher levels of unspliced HIV RNA (Fig. 2A & B). We also determined the total of unspliced and spliced HIV RNA in these transfected cells using qRT-PCR and a pair of primers that were designed to allow detection of both unspliced and spliced HIV RNA [83]. Consistent with the unspliced HIV RNA, more Metformin led to increases in total HIV RNA (Fig. 2C). Furthermore, we performed similar qRT-PCR experiments to determine the total HIV RNA in HIV-infected and Metformin-treated Jurkat. Similar results were obtained (Fig. 2D). To ascertain that Metformin treatment indeed led to increased HIV transcription, we first treated HIV LTR promoter-driven luciferase (Luc) reporter cell line TZM-bl with Metformin and determined the Luc activity. Higher Luc activities were detected with higher concentrations of Metformin (Fig. 3A). We then transfected 293T with HIV LTR-driven Luc reporter plasmid, treated these cells with Metformin, and determined the Luc activity. Similar results were obtained (Fig. 3B). All these results together demonstrated that Metformin treatment led to activation of the HIV LTR promoter transcription.

The HIV LTR promoter is comprised of three distinct regions U3, R, and U5. U3 is further divided into the core promoter, the enhancer, and the modulatory region [89]. R contains trans-activation response element TAR region responsible for Tat-enhanced transcription of full-length of HIV RNA. A number of DNA binding sites have been identified within U3 for cellular transcription factors, including multiple DNA binding sites for transcription factors activating protein 1 (AP1) and nuclear factor kappa B (NF-κB). Thus, we first took advantage of AP1 DNA binding site-driven Luc reporter gene (AP1-Luc), NF-κB DNA binding site-driven Luc reporter gene (NF-κB-Luc), and TATA DNA binding site-driven Luc reporter gene (TATA-Luc) and determined the Luc reporter gene activities in response to Metformin treatment. Lower Luc activities were detected with higher Metformin with AP1-Luc (Supplementary Fig. 3A) and NF-κB-Luc (Supplementary Fig. 3B). In comparison, higher Luc activities were detected with higher Metformin with TATA-Luc (Supplementary Fig. 3C). To further determine the combined effect of Metformin on the HIV LTR core promoter that contains the TATA-box and DNA binding sites for NF-κB and other transcription factors, we performed the HIV LTR core promoter-driven Luc reporter gene assay in the presence of Metformin and found the direct activation of Metformin on the HIV LTR core promoter (Supplementary Fig. 4), suggesting a net enhancement effect of Metformin on the HIV LTR core promoter.

### Increased CREB expression and/or phosphorylation and TBP expression by Metformin and HIV

**Figure 4.**
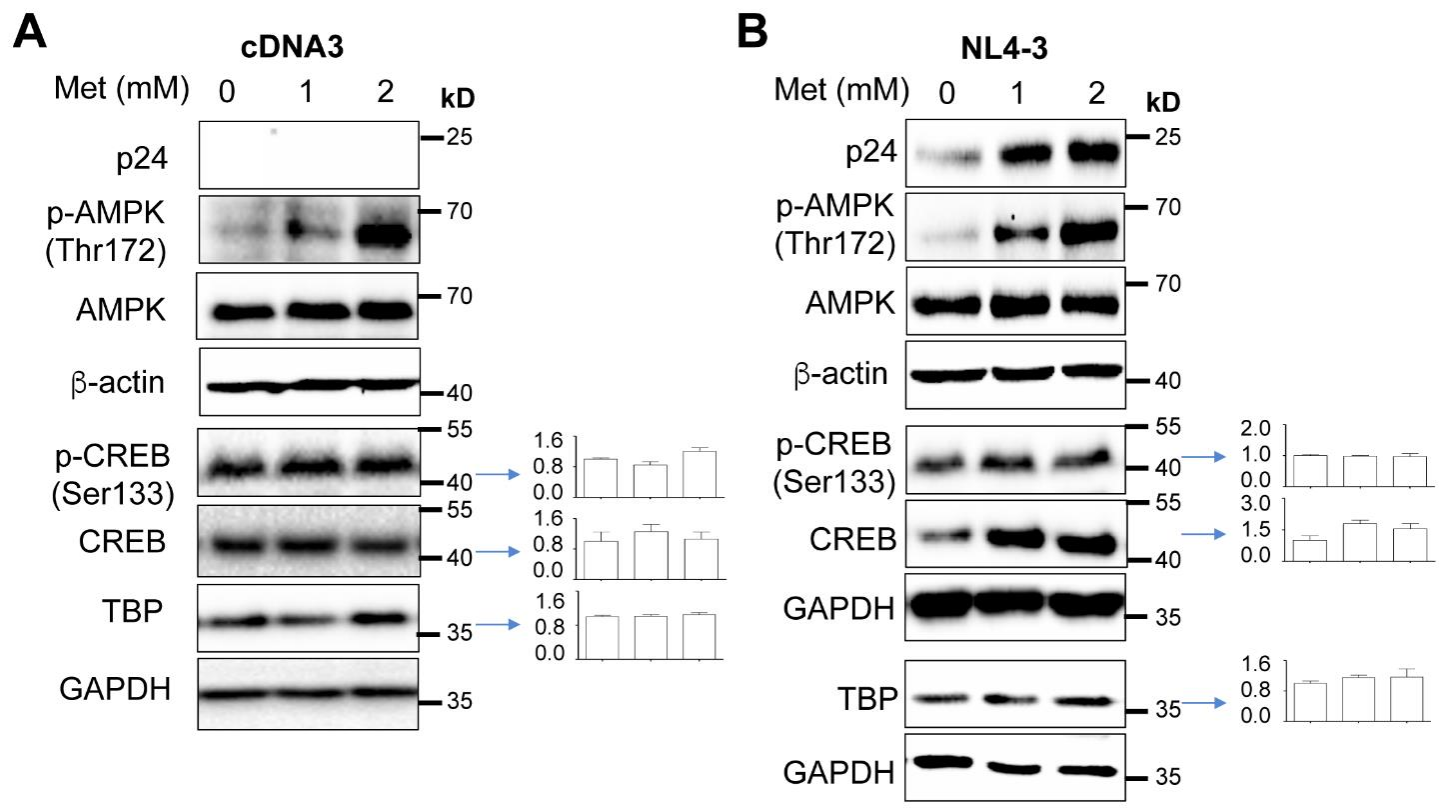
**Effects of Metformin on expression of transcription factors CREB and TBP**. 293T were plated in a 6-well plate at a density of 2 x 10^5^ per well, transfected with 3.5 μg pcDNA3 (A), or pNL4-3 (B), cultured for 16 h, changed fresh medium, added Metformin, and continued to culture for 48 h. The cells were harvested for Western blotting against an anti-p24, p-AMPK, AMPK, p-CREB, CREB, TBP, GAPDH, or β-actin antibody. p-CREB was normalized to CREB, while CREB and TBP expression were normalized to the loading control GAPDH. The data were representative of three independent experiments and Mean ± SD of multiple samples (CREB & TBP, N = 3; p-CREB, N = 2).

**Figure 5.**
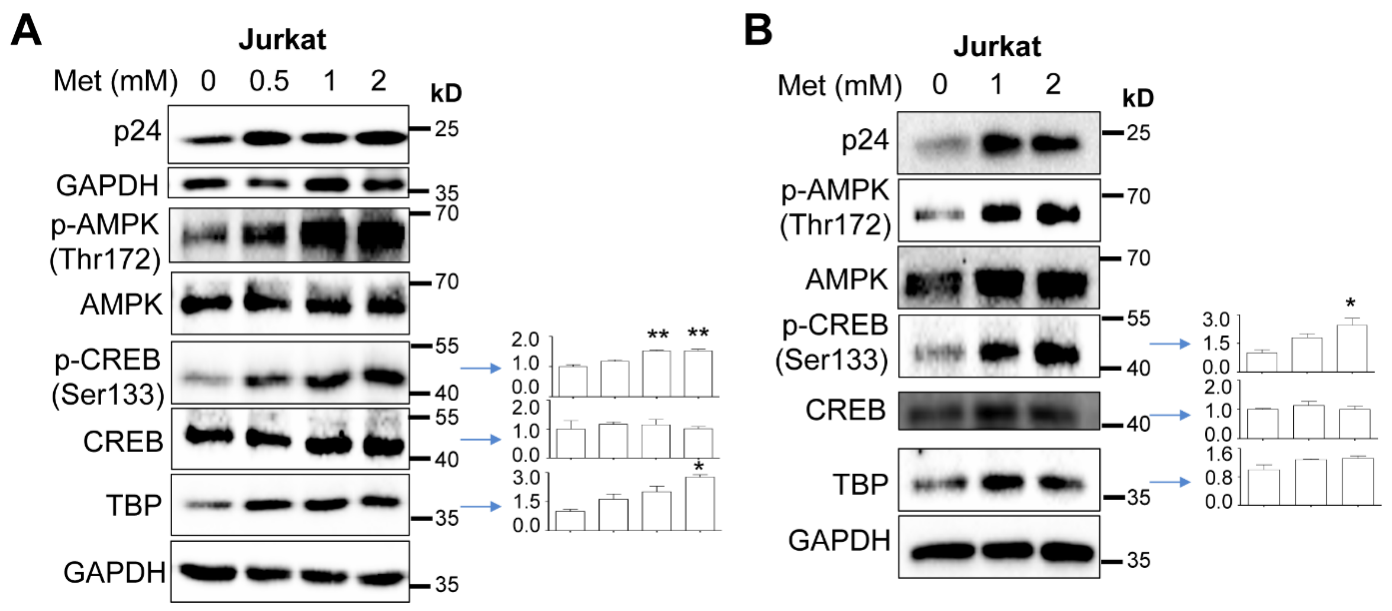
**Effects of Metformin on expression of transcription factors CREB and TBP in the context of HIV infection**. **(A)** Jurkat (1 x 10^6^) were infected with 10,000 cpm-equivalent HIV NL4-3 viruses via spinoculation in the presence of 1 x polybrene, washed with PBS, cultured for 5 days with changes of fresh medium every 48 h, added Metformin, and continued to culture for 72 h. The cells were harvested for Western blotting. **(B)** Jurkat (1 x 10^5^) were infected with 100,000 cpm-equivalent VSV-G-pseudotyped HIV-Luc, added Metformin, and cultured for 72 h. The cells were washed with PBS and collected for Western blotting. p-CREB was normalized to CREB, while CREB, and TBP expression were normalized to the loading control GAPDH. The data were representative of two (A) and three (B) independent experiments.

The similar enhancement effects of Metformin on HIV gene expression, transcription and production and the TATA-Luc reporter gene activities prompted us to focus on the transcription factors that target the TATA-box of the HIV LTR. TATA-binding protein (TBP)/TFIID are known to bind to the TATA-box region, recruit other basal transcription factors to the promoter to form the RNA polymerase II transcription complex, and facilitate transcription initiation and elongation [90]. TBP also binds to cellular transcription factors and viral proteins to activate transcription [91]. Among these transcription factors is CREB, which functions as a dimer upon phosphorylation [92-95] and has multiple cAMP response elements within the HIV LTR promoter and promotes HIV transcription through the cAMP pathway and CREB binding [96]. Thus, we next determined effects of Metformin on CREB expression and phosphorylation and TBP expression using Western blotting. Compared to the pcDNA3 transfection control (Fig. 4A), pNL4-3 transfection showed trends of increases in CREB and TBP expression and slight increases in CREB phosphorylation over Metformin treatment (Fig. 4B). Consistent with previous studies [42, 44, 45, 47, 48, 97-100], Metformin treatment led to AMPK phosphorylation in both pcDNA3 and pNL4-3 transfections, albeit with no significant differences between these two transfections. To determine if these changes would occur in the context of HIV infection, we infected Jurkat with NL4-3, treated them with Metformin, and performed Western blotting. Similar results were obtained except for a more pronounced increase in CREB phosphorylation (Fig. 5A). To further validate these findings, we also performed single-round infection of Jurkat with VSV-G-pseudotyped HIV, treated them with Metformin, and performed Western blotting. Similar to NL4-3 infection, VSV-G-pseudotyped HIV infection showed comparable trends of increases in CREB phosphorylation and TBP expression (Fig. 5B).

### Increased recruitment of phosphorylated CREB and TBP to the HIV LTR promoter by Metformin

**Figure 6.**
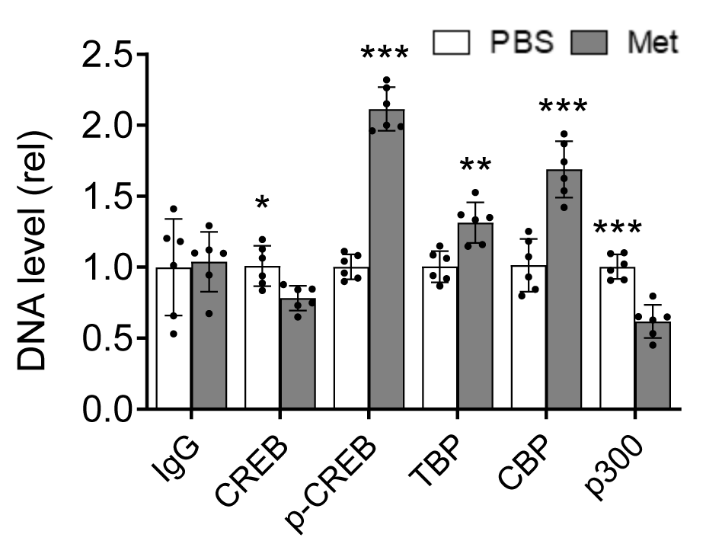
**Effects of Metformin on recruitment of transcription factors CREB and TBP onto HIV LTR promoter**. 293T were plated in a 10 cm cell culture dish at a density of 2x 10^6^ per plate, transfected with 20 μg HIV pNL4-3, cultured for 16 h, replaced with fresh medium, added PBS or Metformin (2 mM), and continued to culture for 48 h. The cells were washed twice with ice-cold PBS and processed for cross-linking and the chromatin immunoprecipitation assay using an anti-CREB, anti-p-CREB, anti-TBP, anti-CBP, anti-p300, or rabbit IgG. The DNA associated with the immunoprecipitates was purified, subjected to qPCR with specific primers spanning the DNA binding sites of each transcription factor, and normalized to corresponding input DNA which was determined using U6 primers. The data were Mean ± SD of six samples (N = 6).

We next determined if Metformin-enhanced CREB phosphorylation and TBP expression would result in their recruitment to the HIV LTR promoter. To this end, we transfected 293T with pNL4-3, treated them with PBS or Metformin, and performed the ChIP assay using specific primers covering the TATA-box region for TBP and the potential DNA binding sites that involve phosphorylated CREB. Corroborated with our previous results (Fig. 4 & 5), Metformin treatment led to detection of more phosphorylated CREB and TBP and less total CREB on the LTR promoter than the PBS treatment control (Fig. 6). In addition, we performed the ChIP assay for transcription co-factors CREB-binding proteins CBP and p300, which interact with both basal transcription factors and transcription activators [101-103]. Metformin treatment led to more recruitment of CBP but less p300 to the HIV LTR promoter compared to the PBS treatment control (Fig. 6).

### Metformin treatment increased HIV gene expression, transcription, and production in human PBMC

**Figure 7.**
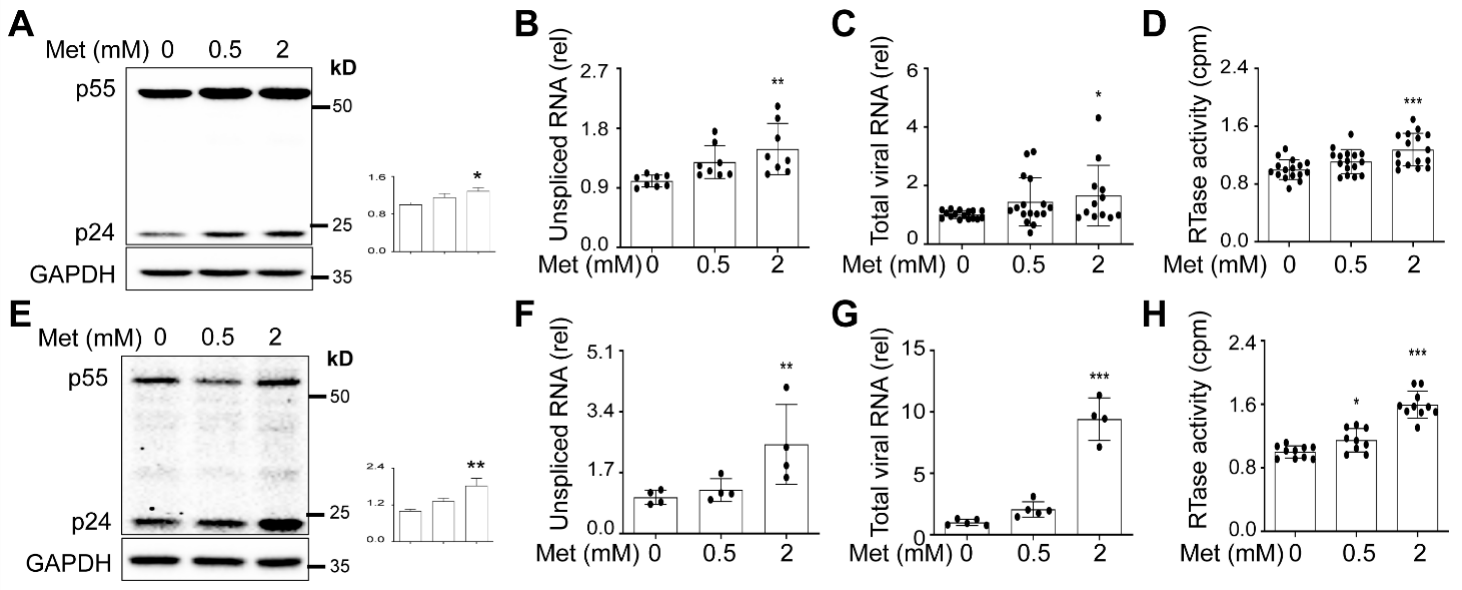
**Effects of Metformin on HIV replication in human PBMC**. **(A-D)** Freshly isolated human PBMC were cultured in the presence of anti-human CD3 antibody (1 μg/ml) and CD28 (2 μg/ml) for 72 h, infected with HIV NL4-3 (MOI: 0.5) by spinoculation as above, washed with fresh medium, added Metformin, and cultured in the human IL-2 (100 IU/ml) for 72 h. The cells were harvested for Western blotting against an anti-p24 or GAPDH antibody, followed by densitometry quantitation for p24 (A), and for total RNA isolation, followed by qRT-PCR for gag-pol RNA (B) or total viral RNA (C). The culture supernatant was collected for the RTase activity assay (D). **(E-H)** Similar experiments were performed except for that human PBMC were infected with HIV NL4-3 (MOI: 0.1) in the presence of Metformin and human IL-2 (100 IU/ml). p24 expression was normalized to the loading control GAPDH (A & E). The RTase activities were normalized to the cellular protein (D & H). The data were representative of six independent experiments (A & E) or Mean ± SD of multiple samples (B, N ≥ 8; C, N ≥ 12; D, N ≥ 16; F & G, N ≥ 4; H, N ≥ 9).

To validate and substantiate our findings obtained from cell lines, we isolated PBMC from healthy donors, cultured them in the presence of anti-human CD3/CD28 antibodies for 3 days, infected them with NL4-3, treated them with Metformin, continued to culture for 3 days, and collected the cells for Western blotting and RNA isolation, and culture supernatant for the RTase assay. Metformin treatment led to increased p24 expression (Fig. 7A), increased unspliced HIV RNA (Fig. 7B) and total HIV RNA (Fig. 7C), and increased HIV production (Fig. 7D) in these cells. We also performed similar experiments in human PBMC with concurrent infection and Metformin treatment. Similar results were obtained except for the fact that more pronounced increases in total HIV RNA were noted with Metformin treatment (Fig. 7E-H).

### Metformin treatment increased CREB phosphorylation and TBP expression and their recruitment to the HIV LTR promoter in HIV-infected human PBMC

**Figure 8.**
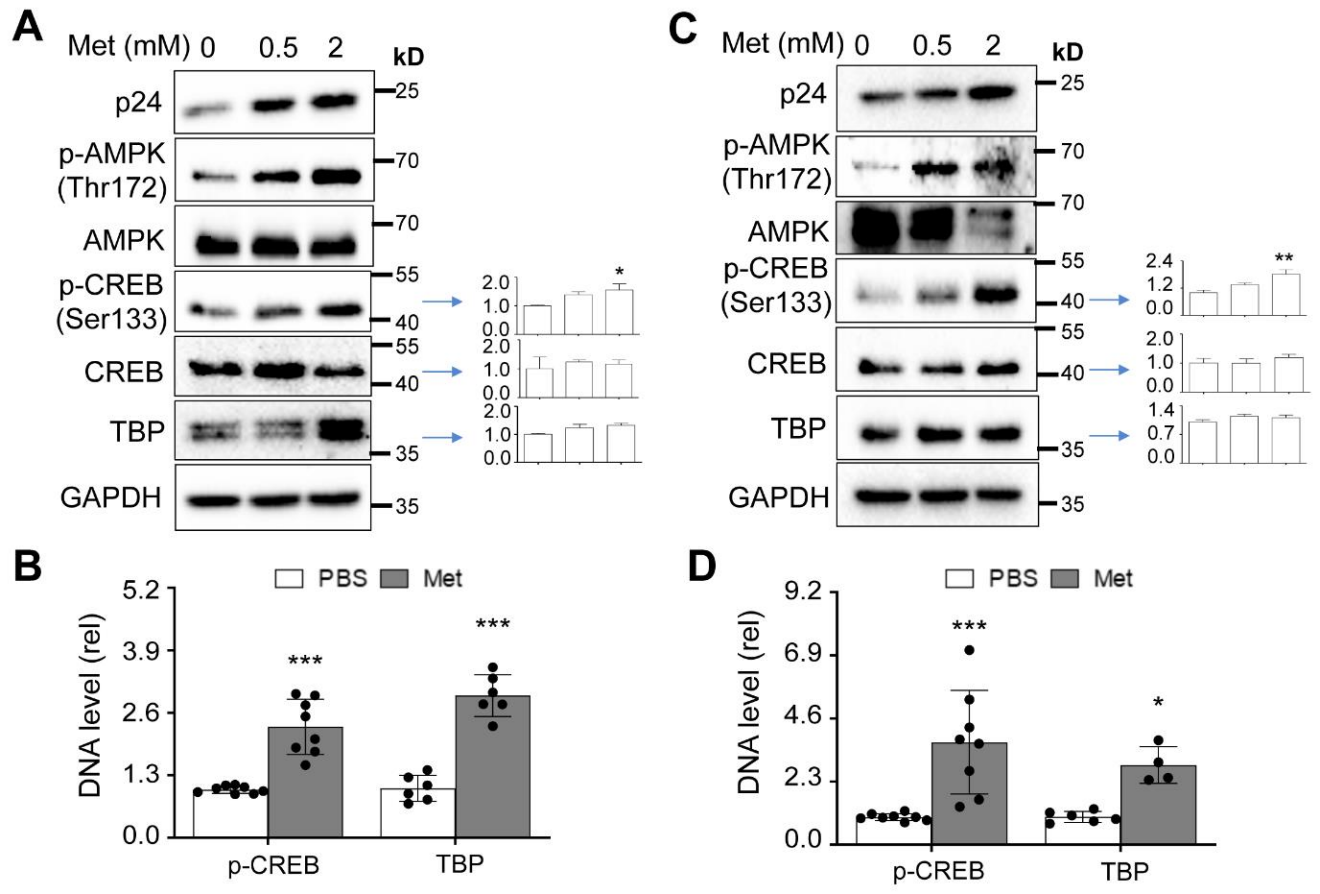
**Effects of Metformin on CREB and TBP expression and their recruitment to the HIV LTR promoter in human PBMC**. Human PBMC from Fig. 7A-D were harvested for Western blotting (A) or ChIP assay (B); and human PBMC from Fig. 7E-H were harvested for Western blotting (C) or ChIP assay (D). p-CREB was normalized to CREB, while CREB, and TBP expression were normalized to the loading control GAPDH (A & C). The ChIP assay readouts were normalized to corresponding input DNA determined with U6 primers (B & D). The data were representative and Mean ± SD of 8 replicates for p-CREB, 3 replicates for CREB, and 4 replicates for TBP (A), and 4 replicates for p-CREB, 4 replicates for CREB, and 6 replicates for TBP (C). p24 and AMPK/p-AMPK were representative of 6 and 2 independent experiments, respectively (A & C). The data were Mean ± SD of multiple samples (B, N ≥ 6; D, N ≥ 4).

We next determined if Metformin treatment would have similar effects on CREB expression and phosphorylation and TBP expression in HIV-infected human PBMC (Fig. 8A-D). Metformin treatment resulted in increased CREB phosphorylation and TBP expression but led to little changes in CREB expression in HIV-infected human PBMC (Fig. 8A). We included AMPK and phosphorylated AMPK as the controls in these experiments and confirmed Metformin-induced AMPK phosphorylation. Then, we performed the ChIP assay and determined the recruitment of phosphorylated CREB and TBP onto the HIV promoter. Metformin treatment led to increased recruitment of phosphorylated CREB and TBP onto the HIV LTR promoter in these cells (Fig. 8B). In addition, we also performed Western blotting and the ChIP assay using the HIV-infected human PBMC derived from Fig. 7E-H and obtained similar results in this infection setting (Fig. 8C & D). The enhancement effects of Metformin on the HIV LTR promoter and transcription prompted us to investigate whether Metformin would increase HIV transcription and production from several previously characterized HIV latent cells with a low level and persistent HIV replication. We first treated HIV-infected latent CD4+ T lymphocytic cell line J1.1 with Metformin and determined HIV p24 expression using Western blotting and HIV RNA expression by qRT-PCR. More p24 expression was detected in the cells treated with more Metformin (Supplementary Fig. 5A). In the meantime, more unspliced HIV RNA and HIV total RNA were detected in the cells treated with more Metformin (Supplementary Fig. 5B & C). Moreover, more phosphorylated CREB was detected in the cells treated with more Metformin (Supplementary Fig. 5D). We also performed similar experiments with HIV-infected latent promonocytic cell line U1. A general trend of increased p55 expression, HIV RNA expression, and phosphorylated CREB was observed, albeit in a slightly different kinetics from J1.1 (Supplementary Fig. 6A-D). Similar results were obtained from another HIV-infected latent CD4+ T lymphocytic cell line ACH-2 (Supplementary Fig. 7A & B) and an HIV-infected latent Jurkat cell line we established using HIV reporter viruses NLGi (Supplementary Fig. 7C). These results together indicate that Metformin treatment enhanced HIV transcription and gene expression and was associated with increased CREB phosphorylation.

### 666-15 treatment significantly abrogated Metformin-enhanced HIV gene expression

**Figure 9.**
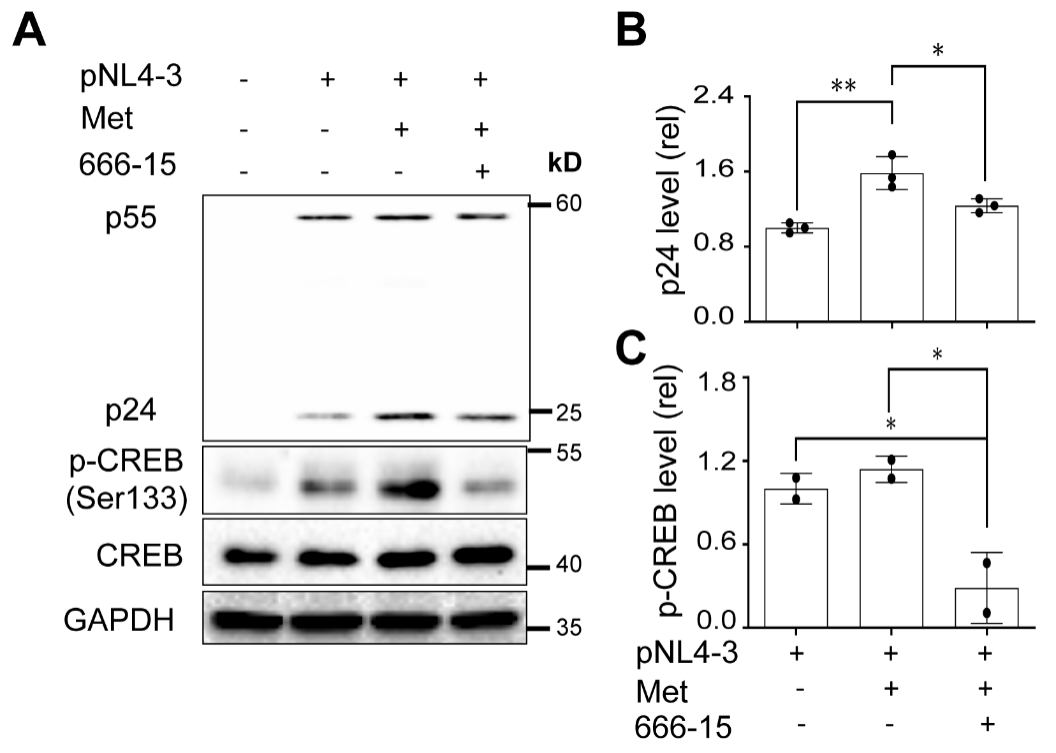
**Effects of 666-15 on HIV gene expression in the presence of Metformin**. 293T were plated at a 6-well cell culture plate at a density of 2 x 10^5^ per well, transfected with 3.5 μg pcDNA3, or pNL4-3, cultured for 16 h, changed medium, treated with Metformin (0.5 mM) or Metformin (0.5 mM) plus 666-15 (0.5 µM) for 48 h. PBS (Metformin solvent) was added as a treatment control for Metformin. DMSO (666-15 solvent) was added as a treatment control for 666-15. The cells were harvested for Western blotting (A), followed by densitometry quantitation for p24 (B) and p-CREB (C), and normalized to CREB. The data were representative of three independent experiments (A) and Mean ± SD of multiple samples (B, N = 3; C, N = 2). The results were analyzed by one-way ANOVA followed by a post-hoc Tukey test.

To substantiate these findings and ascertain the mechanisms responsible for Metformin-enhanced HIV gene expression, we took advantage of 666-15, a potent and selective inhibitor of CREB activation [104-106] and investigated the effects of this inhibitor on Metformin-enhanced HIV gene expression. As expected, 666-15 treatment led to inhibition of CREB phosphorylation (Fig. 9A & C). In the meantime, it also led to a significantly lower level of p24 expression in 293T treated with Metformin than those treated with Metformin only (Fig. 9A & B), further ascertaining that CREB activation is the major mechanism responsible for Metformin-enhanced HIV gene expression.

## DISCUSSION

In this study, we first transfected 293T with pNL4-3 or infected Jurkat and human PBMC with NL4-3 and treated the cells with Metformin. We showed that Metformin treatment increased HIV gene expression and transcription in these cells. Due to the cytotoxicity, we performed subsequent mechanistic experiments using Metformin at a concentration of up to 2 mM and further normalized the results to the total cellular protein or cell count. Metformin and its subsequent activation of AMPK have been shown to have diverse effects on infection of various pathogens including viruses, bacteria, and parasites [107]. The effects could be positive or negative, depending on the pathogens. Metformin/AMPK activation promotes replication of viruses such as rotavirus [108] and is implicated in pathogenesis of herpes simplex virus type 1 [109], and Epstein-Barr virus [110], or inhibits replication of viruses such as hepatitis C virus [111, 112], Zika virus [113], and Dengue virus [114-116]. In case of HIV, AMPK activation is involved in epigallocatechin-3-O-gallate-induced inhibition of Tat transactivation activity on the HIV LTR promoter [117]. AMPK activation is also involved in HIV infection-induced energy deficit and metabolic dysfunction in the context of cocaine use [118]. In contrast to our findings, Metformin has recently been shown to inhibit HIV replication in primary human CD4+ T cells and Jurkat [119]. The main differences between our study and the above-mentioned study appears to be that we observed apparent anti-proliferative effects of Metformin on all the cells tested at the same concentrations at which the other study did not notice the anti-proliferative effects of Metformin, although these two studies normalized the readouts to the cell number or total cellular proteins (email communications between Drs. He and Guo on April 16, 2021). Interestingly, the very same study also showed a strong positive correlation between HIV replication and the nucleotide-binding domain and leucine-rich repeat containing receptor X1 transcript levels, which was significantly increased in primary CD4+ T cells when treated with Metformin. These apparent counterintuitive results were interpreted as a compensatory mechanism [119].

The discordance between pharmacological Metformin concentrations in human plasma and *in vivo* animal studies (0.5-30 μM) and supra pharmacological Metformin concentrations (> 1 mM) *in vitro* cell culture studies has been a common subject of debate in the field of Metformin research since Metformin was discovered about 70 years ago. Metformin is known to inhibit gluconeogenesis *in vivo* through inhibition of mitochondrial respiratory chain complex I and activation of AMPK signaling. However, none of these mechanisms have consistently been reproduced in cell cultures when Metformin concentrations are < 1 mM. A number of possibilities have been attributed for this discordance.

Among them are lack of Metformin-binding (retaining) proteins in plasma and subsequent high excretion of Metformin through kidney because of highly positive charge of Metformin, selective uptake and accumulation of Metformin in different organs, tissues and cells leading to much higher Metformin working concentrations (than those detected in plasma), different expression of Metformin transporters such as organic cation transporter 1 between cells in organs and tissues *in vivo* and cell lines *in vitro*, and different glucose concentrations between cells *in vivo* and cell cultures *in vitro.* Thus, the selection of Metformin concentrations in our experiments was based on and consistent with those in the literature [45, 97, 99, 120-122]. It is also important to note that the accumulation of Metformin in tissues is several times higher than the reported concentrations in the blood [48, 123-125], and that the accumulation of Metformin in cell lines are only 10-15% of Metformin in the culture medium [126, 127].

In the study, we also showed that Metformin re-activated HIV in three lymphocytic cell lines, and one promonocytic cell line. In a 12-week clinical trial involving 13 non-diabetic antiretroviral-suppressed HIV patients (HIV viral load in the blood < 40 copies/ml), Metformin did not show any remarkable effects on the reservoir size of HIV-infected latent CD4+ T cells in the blood but decreased residual HIV transcription in CD4+ T cells within the colons of 8 HIV-infected individuals, while increased HIV transcription in CD4+ T cells within the colons of 4 other HIV-infected individuals [61, 62]. Memory CD4+ T cells constitute the major HIV reservoir in HIV-infected individuals under antiretroviral therapy [128, 129]. Metformin treatment has been shown to cause fewer HIV-infected memory CD4+ T reservoirs, which co-express negative immune checkpoint receptors in HIV-infected individuals [63]. Consistent with increased HIV transcription and re-activation of HIV from latency, we showed that Metformin treatment led to increases of the HIV LTR promoter activity. A variety of cellular mechanisms are involved in the establishment and maintenance of HIV latency [130]. Among them is stable repression of the chromatin at the HIV LTR promoter region [130, 131], which is primarily controlled by the activities of histone acetylases/deacetylases and lack or sequestration of transcription factors and co-activators/co-repressors [132, 133]. The findings from all the clinical trial studies as well as from the current study together indicate that Metformin treatment, when used in combination with ART, could help decrease the size of HIV reservoirs in HIV-infected individuals with or without type 2 diabetes, facilitate eventual elimination of HIV reservoirs, and achieve HIV cure.

We then determined the underlying molecular mechanisms whereas Metformin treatment augmented HIV transcription by investigating the direct effects of Metformin on the activities of the promoters containing individual DNA-binding sites of transcription factors within the HIV LTR promoter. Consistent with previous studies [134-137], we showed that Metformin treatment inhibited NF-κB activity. In addition, we showed that Metformin treatment inhibited AP-1 activity in human embryonic kidney epithelial cell line 293T, while Metformin has been found to enrich AP-1 transcription factor and its regulatory gene network in normal human fibroblasts [138]. This discrepancy may be attributed to the cell-type difference of AP-1 expression and activity between normal human fibroblasts and 293T that were used in our study. Furthermore, we noticed that Metformin treatment led to increased TATA-box transcription activity, which we believe may account for, at least in part, the augmentative effects of HIV transcription. Nevertheless, we performed Western blotting and determined effects of Metformin on CREB expression and phosphorylation and TBP expression. We showed that Metformin treatment resulted in increased CREB expression and phosphorylation and TBP expression in the context of HIV. There are several cAMP-responsive element (CRE) sequences within the HIV LTR promoter, which is the binding site for phosphorylated and dimerized CREB [139-141]. One CRE is located immediately upstream of the transcription start site (+1) of the HIV LTR promoter. Metformin has been shown to decrease CREB phosphorylation and the CRE activity in epithelial cell line MCF-7 [142] but to increase CREB phosphorylation in neuroblastoma cell line SH-SY5Y [143, 144], suggesting again that Metformin effects on CREB phosphorylation is cell type-dependent.

Lastly, we demonstrated that Metformin treatment led to increased recruitment of p-CREB and TBP to the HIV LTR promoter. Phosphorylation of CREB at Ser-133 leads to the recruitment of CBP to CRE through direct interaction and complex formation between phosphorylated CREB and CBP [145]. The tripartite interactions between phosphorylated CREB, CBP, and RNA Pol II as well as bipartite nexus between CREB and TFIID complex have been well demonstrated [146]. The recruitment of CBP by phosphorylated CREB followed by RNA Pol II engagement does not suffice to trigger transcription, and that activated CREB further mediates the recruitment of TFIID as a requirement for transcription induction of the signal-reliant target genes [146]. Interestingly, Metformin in our study did not change TBP expression in non-HIV transfected cells, but slightly increased TBP expression in HIV transfected/infected cells. Besides the increased expression of phosphorylated CREB and TBP, the significant recruitment of these factors alongside CBP to the HIV LTR by Metformin in the context of HIV infection was quite noteworthy. A recent study has indeed shown that activation of the cAMP-PKA-CREB signaling pathway results in enhanced HIV LTR promoter transcription and HIV replication [140]. Importantly, we showed that the inhibition of Metformin-enhanced CREB activation by CREB activation inhibitor 666-15 resulted in a marked decrease in HIV gene expression. These data provide additional mechanistic evidence to support the important roles of CREB activation in Metformin-enhanced HIV transcription and gene expression. Nevertheless, the molecular mechanisms by which Metformin treatment leads to AMPK-independent CREB phosphorylation remains to be investigated.

## Supplementary Materials

The Supplementary data can be found online at: www.aginganddisease.org/EN/10.14336/AD.2023.0705.


