# Supplementary material for: Metformin Treatment Leads to Increased HIV Transcription and Gene Expression through Increased CREB Phosphorylation and Recruitment to the HIV LTR Promoter
Source: Aging Dis. 2024 Apr 1;15(2):831–50. doi: 10.14336/AD.2023.0705 (PMC10917544; doi:10.14336/AD.2023.0705)
Supplement: Supplementary file 1 [file AD-15-2-831-s.pdf]

SUPPLEMENTARY DATA

Metformin Treatment Leads to Increased HIV Transcription and Gene Expression through Increased CREB Phosphorylation and Recruitment to the HIV LTR Promoter

Sahar Rezaei, Khalid A. Timani, Johnny J. He

© 2023. Rezaei S et al. Published online at http://www.aginganddisease.org/EN/10.14336/AD.2023.0705

SUPPLEMENTARY DATA

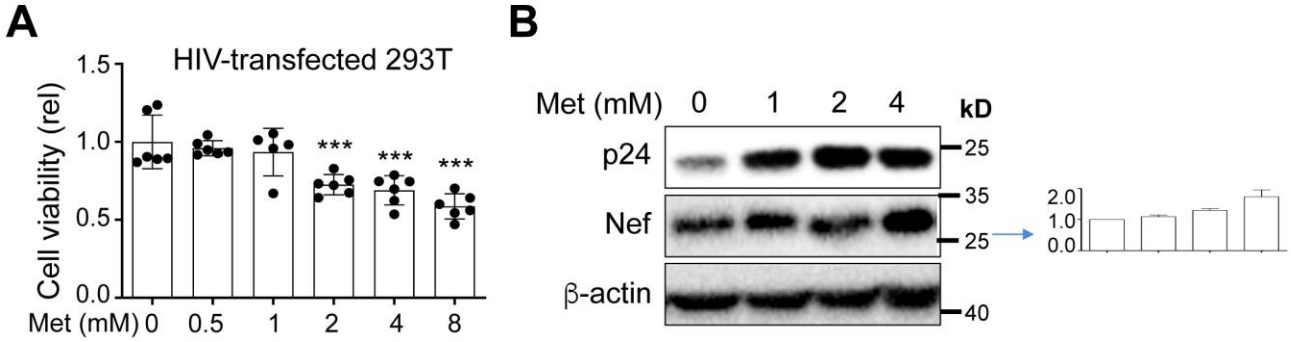

Supplementary Figure 1. Effects of Metformin on cell viability in the context of HIV and viral Nef expression. 293T were plated in a 24-well plate at a density of 0.6 x 10^5 per well, cultured for 24 h, transfected with 0.8 μg pNL4-3, continued to culture for 16 h, replaced the medium, treated with either PBS or Metformin for 48 h, and processed for the MTT assay (**A**), or for Western blotting against an anti-Nef, anti-p24 or anti-β-actin antibody (**B)**. Nef expression was quantitated by densitometry, normalized to β-actin, and expressed in fold-change in reference to the first sample without Metformin treatment. The data were Mean ± SD of six samples (**A**, N = 6) or representative of two independent experiments (**B**).

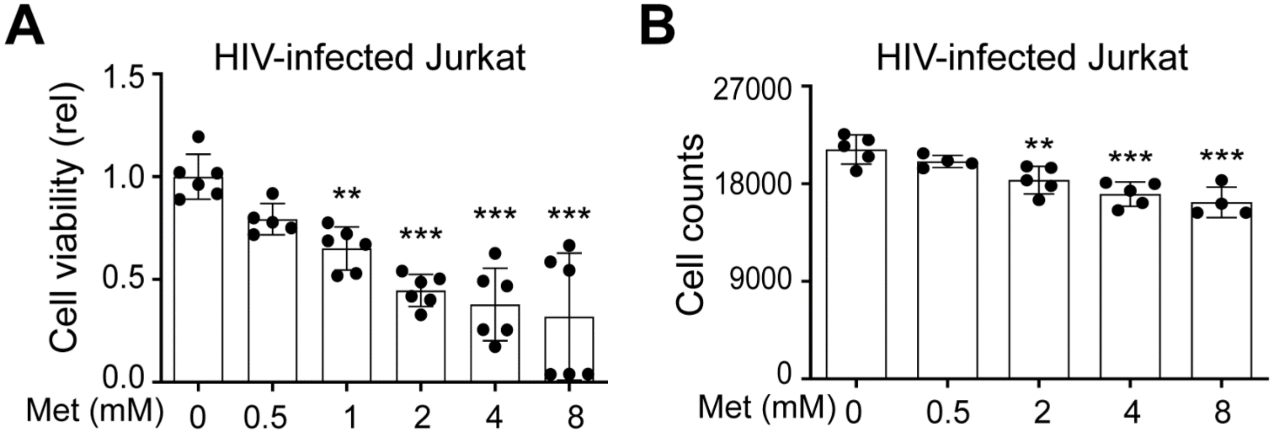

Supplementary Figure 2. Effects of Metformin on viability and proliferation of HIV-infected Jurkat. A. HIV-infected Jurkat were plated in a 24-well plate at the density of 0.6 x 10^5 per well, cultured for 24 h, and treated with Metformin for 48 h. The medium was removed and replaced with a fresh RPMI 1640 medium and processed for the MTT assay. **B.** HIV-infected Jurkat were plated in a 96-well white tissue culture plate with a clear bottom at a density of 1 x 10^4 per well, cultured for 24 h, treated with or without Metformin for 48 h, and processed for the fluorometric cell proliferation assay. The results were expressed and statistically analyzed using the PBS control as a reference. The data were Mean ± SD of multiple samples (**A**, N = 6; **B**, N = 5).

© 2023. Rezaei S et al. Published online at http://www.aginganddisease.org/EN/10.14336/AD.2023.0705

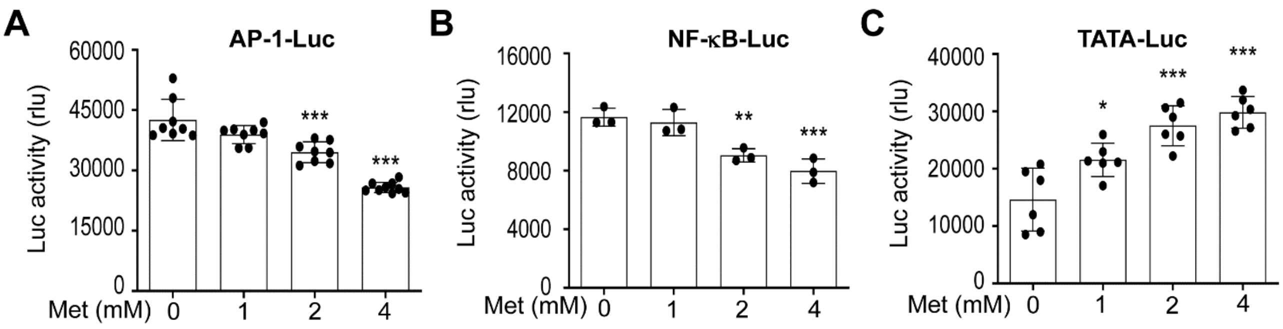

Supplementary Figure 3. Effects of Metformin on the Ap-1 and NF-κB binding sites-driven, and TATA Box-driven promoter activities. 293T were plated in a 12-well plate at a density of 1 x 10^5 cells/well, transfected with 0.15 µg Ap-1-Luc (**A**), 0.3 µg NF-κB-Luc (**B**), or 30 ng TATA-Luc (**C**) adjusted the final DNA concentration for transfection to 1.5 µg using pcDNA3 cultured for 16 h, changed with fresh medium, added Metformin, continued to culture for 48 h, and harvested for the Luc reporter gene assay. The Luc activity was normalized to the cellular proteins. The data were Mean ± SD of multiple samples (**A**, N = 8; **B**, N = 3; **C**, N = 6).

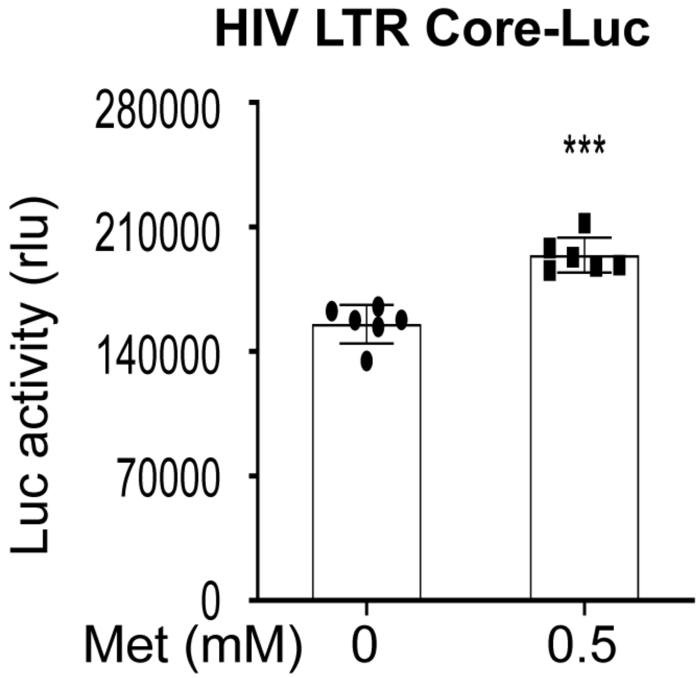

Supplementary Figure 4. Effects of Metformin on the HIV LTR core promoter-driven promoter. 293T were plated in a 12-well plate at a density of 1 x 10^5 cells/well, transfected with 0.125 µg LTR core promoter-driven Luc reporter construct plus 1.5 µg pcDNA3, cultured for 16 h, replaced with fresh medium, and treated with Metformin. PBS was added as the treatment control for Metformin. Cells were harvested for the Luc reporter gene assay 48 h post transfection. The data were Mean ± SD of six samples (N = 6).

© 2023. Rezaei S et al. Published online at http://www.aginganddisease.org/EN/10.14336/AD.2023.0705

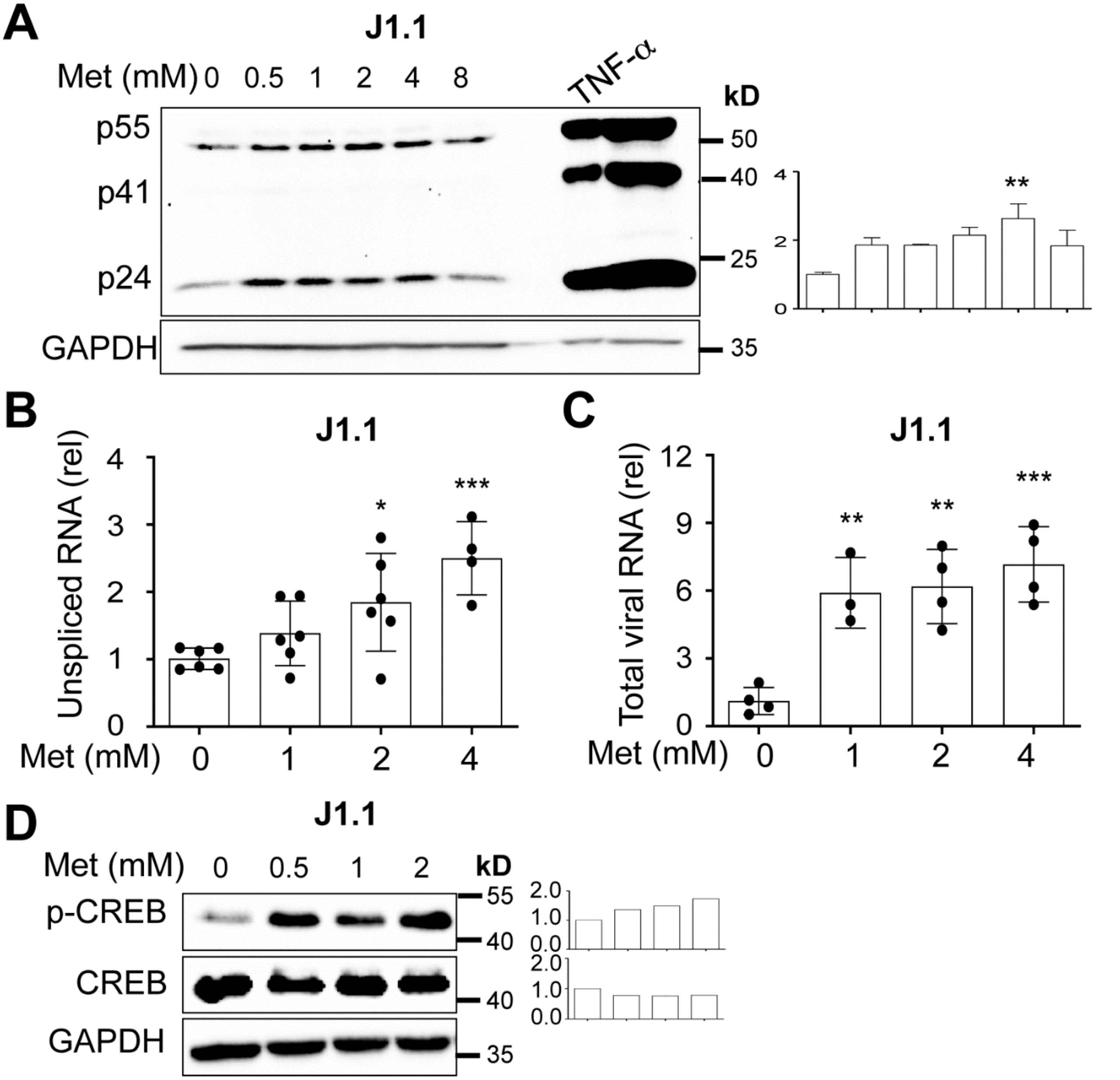

Supplementary Figure 5. Effects of Metformin on HIV p24 expression, transcription, CREB expression and phosphorylation in J1.1 cells latently infected with HIV. J1.1 were plated in a 6-well plate at a density of 2 x 10^5 per well, added Metformin, cultured for 72 h, and harvested for Western blotting against an anti-p24 antibody followed by densitometry quantitation for p24 (**A**), or an anti-p-CREB, CREB, or GAPDH antibody followed by densitometry quantitation for CREB and p-CREB (**D**) or RNA isolation followed by qRT-PCR (**B & C**). TNF-α (10 ng/ml) was included as a positive control. p24 and CREB expression levels were normalized to the loading control GAPDH, and p-CREB was normalized to CREB. The data were representative of three (**A**) and two (**D**) independent experiments or Mean ± SD of multiple samples (**B**, N ≥ 4; **C**, N ≥ 3).

© 2023. Rezaei S et al. Published online at http://www.aginganddisease.org/EN/10.14336/AD.2023.0705

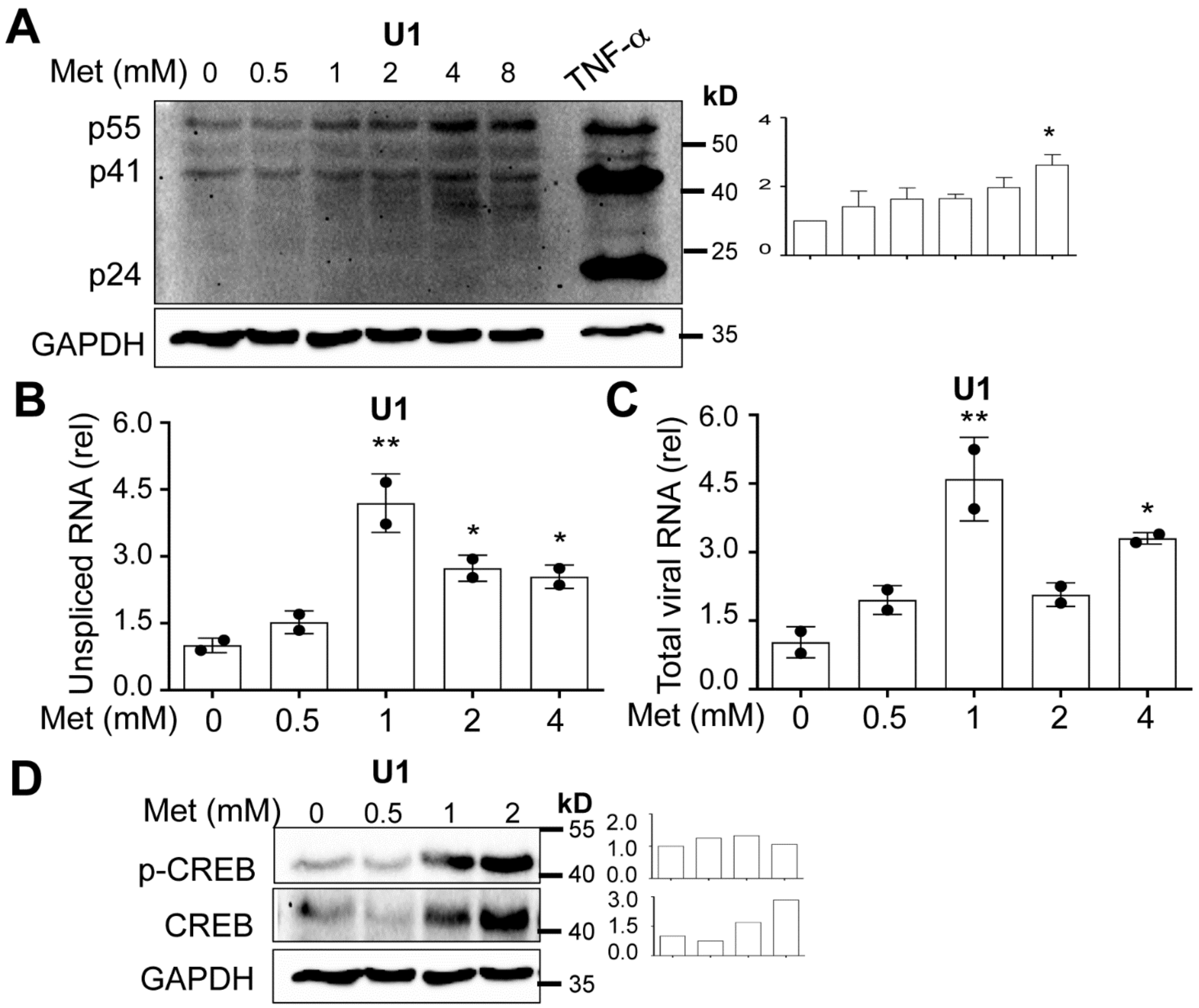

Supplementary Figure 6. Effects of Metformin on HIV p24 expression, transcription, CREB expression, and phosphorylation in U1 cells latently infected with HIV. U1 were plated in a 6-well plate at a density of 2 x 10^5 per well, added Metformin, cultured for 72 h, and harvested for Western blotting against an anti-p24 antibody and graphed for the relative expression of p55 (**A**), or an anti-p-CREB, CREB, or GAPDH antibody followed by densitometry quantitation for CREB and p-CREB (**D**) or RNA isolation followed by qRT-PCR (**B & C**). TNF-α (10 ng/ml) was included as a positive control. p55 and CREB protein expression levels were normalized to the loading control GAPDH while p-CREB was normalized to CREB. The GAPDH in panels **A** & **D** were from the same Western blot of the same samples and the same experiments. The data were representative of two independent experiments (**A** & **D**) or Mean ± SD of two samples (**B** & **C**, N = 2).

© 2023. Rezaei S et al. Published online at http://www.aginganddisease.org/EN/10.14336/AD.2023.0705

SUPPLEMENTARY DATA

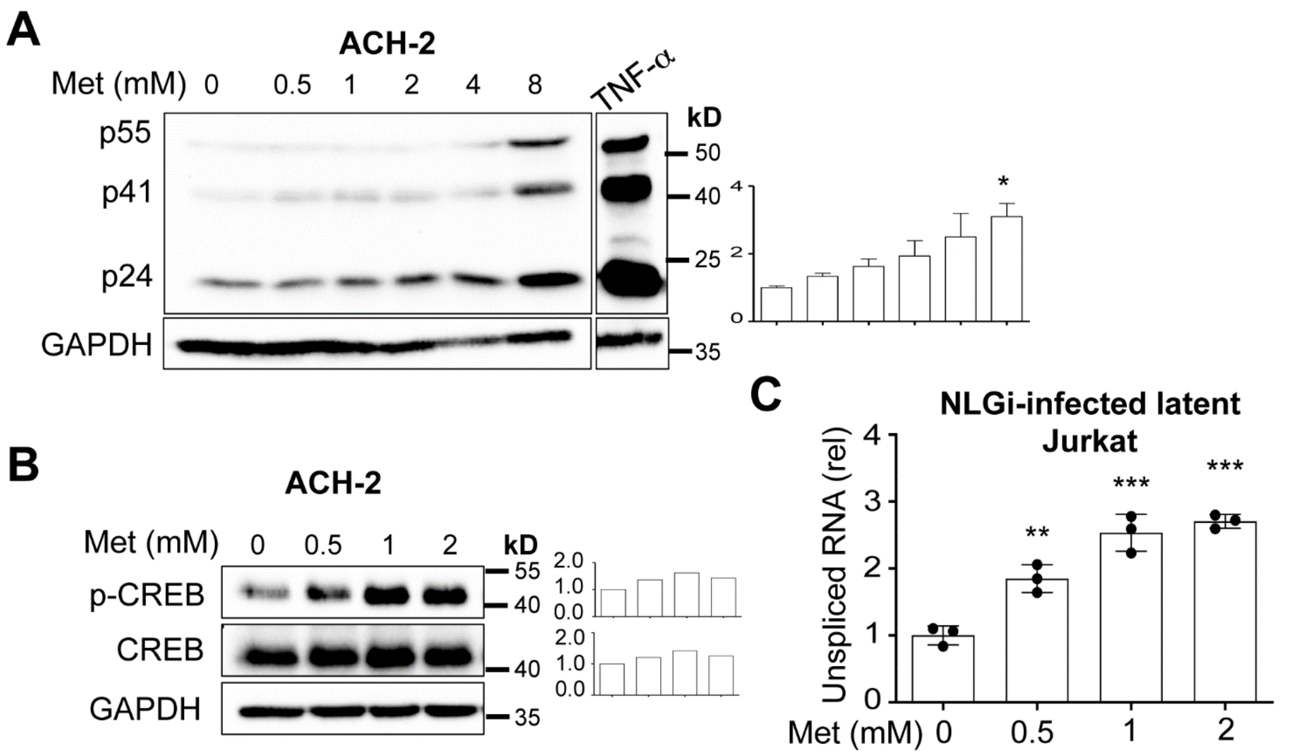

Supplementary Figure 7. Effects of Metformin on HIV p24 expression, CREB expression and phosphorylation in ACH-2 latently infected with HIV and HIV transcription in NLGi-infected latent Jurkat. **A** & **B.** ACH-2 were plated in a 6-well plate at a density of 2 x 10^5 per well, treated with Metformin, cultured for 72 h, and harvested for Western blotting against an anti-p24 antibody (**A**), or an anti-p-CREB, CREB, or GAPDH antibody (**B**). TNF-α (10 ng/ml) was included as a positive control. p24 and CREB protein expression levels were normalized to the loading control GAPDH while p-CREB was normalized to CREB. **C**. Jurkat ($1x10^6$) were infected with 10,000 cpm-equivalent NLGi. The infected Jurkat were cultured and maintained for 63 days while the media was refreshed every three days to obtain NLGi-infected latent cells. These cells were treated with PBS or Metformin for 72 h, followed by TRIzol RNA extraction and Real-Time qRT-PCR to measure gag-pol transcripts. The data were representative of three (**A**) and two (**B**) independent experiments or Mean ± SD of three samples (**C**, N = 3).

© 2023. Rezaei S et al. Published online at http://www.aginganddisease.org/EN/10.14336/AD.2023.0705
